# Rtpca: an R package for differential thermal proximity coaggregation analysis

**DOI:** 10.1093/bioinformatics/btaa682

**Published:** 2020-07-27

**Authors:** Nils Kurzawa, André Mateus, Mikhail M Savitski

**Affiliations:** European Molecular Biology Laboratory, Genome Biology Unit, 69117 Heidelberg, Germany; Candidate for Joint PhD Between EMBL and Heidelberg University, Faculty of Biosciences, 69120 Heidelberg, Germany; European Molecular Biology Laboratory, Genome Biology Unit, 69117 Heidelberg, Germany; European Molecular Biology Laboratory, Genome Biology Unit, 69117 Heidelberg, Germany

## Abstract

**Summary:**

Rtpca is an R package implementing methods for inferring protein–protein interactions (PPIs) based on thermal proteome profiling experiments of a single condition or in a differential setting via an approach called thermal proximity coaggregation. It offers user-friendly tools to explore datasets for their PPI predictive performance and easily integrates with available R packages.

**Availability and implementation:**

Rtpca is available from Bioconductor (https://bioconductor.org/packages/Rtpca).

**Supplementary information:**

Supplementary data are available at *Bioinformatics* online.

## 1 Introduction

Many crucial cellular processes are executed by ensembles of stably or dynamically associated proteins. Systematic profiling of protein–protein interactions (PPIs) has been performed using cell extracts and combining bait prey enrichment or coelution strategies with mass spectrometry (Ewing *et al.*, 2007; Gatto *et al.*, 2014; Heusel *et al.*, 2019; Roux *et al.*, 2012). However, many protein interactions are disrupted even when using very mild lysis conditions (Perrin *et al.*, 2020; Tan *et al.*, 2018). Recently, it has become possible to systematically measure thermal stability of proteins inside living cells using thermal proteome profiling (TPP) (Franken *et al.*, 2015; Savitski *et al.*, 2014). Interacting proteins generally display thermal coaggregation, which can be measured by similarity of thermal profiles obtained by TPP of two or more proteins—a concept termed thermal proximity coaggregation (TPCA) (Tan *et al.*, 2018). This method can inform on the protein interaction state inside the cell, but no freely available and user-friendly software implementation for this analysis exists.

## 2 The Rtpca package

Here, we present an open-source R package—Rtpca—for the analysis of coaggregation of proteins based on TPP experiments (Mateus *et al.*, 2020; Savitski *et al.*, 2014). Our package not only implements the TPCA procedure described by Tan *et al.* (2018)—including functions to easily generate plots, such as receiver operating characteristic (ROC) curves, and functions to test for thermal coaggregation of protein pairs or protein complexes—but also offers an improved method for comparing PPIs across different conditions. Rtpca integrates seamlessly with the existing R infrastructure, by e.g. enabling the direct handling of data structures imported with the TPP R Bioconductor package (Franken *et al.*, 2015).

## 3 Differential testing

**Fig. 1.**
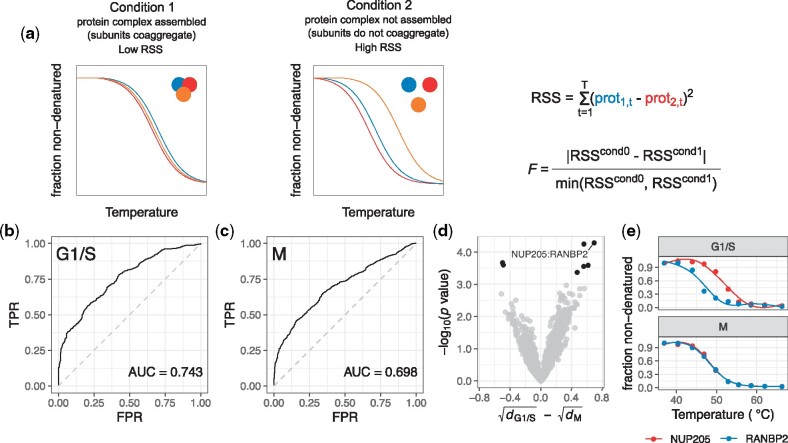
Analysis performed with Rtpca on the TPP dataset by Becher *et al.* (2018) profiling the cell cycle phases M and G1/S. (**a**) Schematic illustrating the principle of Rtpca. RSS, residual sum of squares. (**b**) ROC curve for the G1/S TPP dataset of HeLa cells obtained by ranking groups of proteins by their Euclidean distance of their melting curves. True positive events are considered proteins part of the same complex (Ori *et al.*, 2016). TPR, true positive rate; FPR, false-positive rate. (**c**) Same as (b) but for the TPP dataset obtained in synchronized HeLa cells arrested in M phase. (**d**) Volcano plot visualizing protein pairs within complexes tested for differential thermal coaggregation between both datasets. *d*: average Euclidean melting curve distance of protein pairs in G1/S or M phase, respectively. (**e**) Melting curves of an example protein pair found to be differentially coaggregating in M versus G1/S phase

The method suggested by Tan *et al.* (2018) computes average Euclidean distances between two or more interacting proteins and compares the obtained average distances with those obtained by the same procedure from randomly chosen, equally sized groups of proteins. We implemented this method for performing TPCA in a single condition. For testing for differential PPIs in two conditions, Tan *et al.* (2018) compared the Euclidean distance of melting curves of two annotated protein interactors with randomly drawn protein pairs. Instead, we test for differential thermal coaggregation of two proteins *i* and *j*, annotated or suspected to be potential interactors, across two conditions c={0,1} by computing an *F* statistic which does not only consider the difference between melting profiles of two protein pairs across two conditions, but also incorporates whether the two proteins do indeed coaggregate in one of the conditions (Fig. 1a). This was done by first computing the residual sum of squares (RSS) for each condition *c* with ${\text{RSS}}^{c}=\sum_{t=1}^{T}{\left(y_{i,t}^{c}-y_{j,t}^{c}\right)}^{2}$, where $y_{i,t}$ and $y_{j,t}$ are median fold changes (from replicates) at each temperature *t* compared to the lowest temperature *t*_1_ for protein *i* and *j*, respectively.

An *F* statistic for each protein pair *i*, *j* was then determined with

(1) $$F=\frac{\left|{\text{RSS}}^{0}-{\text{RSS}}^{1}\right|}{\text{min}({\text{RSS}}^{0},{\text{RSS}}^{1})}.$$

To assess significance, obtained *F* statistics were compared to the distribution of $F^{*}$ statistics generated by performing the above-described procedure for 10^6^ randomly drawn pairs of proteins $i^{*}$ and $j^{*}$. An empirical *P*-value was then computed for each threshold *θ* of observed F-statistics with:

(2) $$\hat{p}(\theta )=\frac{\#\{F^{*}\geq \theta \}}{\#\{F^{*}\}}.$$

## 4 Using Rtpca to detect differential PPIs

To showcase how Rtpca can be used to analyze TPP datasets to detect changes in PPIs across different conditions, we re-analyzed a dataset of TPP experiments in HeLa cells at the cell cycle stages G1/S and M (Becher *et al.*, 2018). We found that datasets of both cell cycle phases showed a high predictive power for recovering annotated protein complexes (Ori *et al.*, 2016) based on melting curve similarity of their protein members (Fig. 1b and c). Performing TPCA in the individual datasets, using the Rtpca package function runTpca(), revealed significant coaggregation of several protein complexes in both conditions [e.g. Nuclear pore complex (NPC)], specifically in G1/S phase (e.g. RNA polymerase III core complex) and specifically in M phase (e.g. Cohesin complex). Next, we performed a differential TPCA analysis [package function runDiffTpca()] considering all PPIs within all protein complexes found to significantly coaggregate in either of the cell cycle stages. This analysis revealed several significant differential coaggregating PPIs between the two conditions (Fig. 1d). Most strikingly, NUP205, an inner ring nucleoporin, and RANBP2, a nucleoporin peripherally associated to the NPC on the cytosolic site, were found to coaggregate in M phase, but showed distinct melting curves in G1/S phase (Fig. 1e). This pattern could be related to the involvement of RANBP2 in nuclear envelope breakdown, including the disassembly of the NPC during mitosis (Prunuske *et al.*, 2006).

## 5 Conclusion

We present Rtpca an R package implementing different tools to assess PPI states based on TPP datasets. In addition to providing a user-friendly implementation of previously suggested methods, we provide a method for testing for differential PPIs across treatment or perturbation conditions. We exemplify the application of our package on a recently published dataset performing TPP at different phases of the cell cycle (Becher *et al.*, 2018). Details on the analysis are available at https://github.com/nkurzaw/Rtpca_analysis.

## Funding

This work was supported by the European Molecular Biology Laboratory (EMBL). N.K. was supported by a fellowship of the EMBL International PhD programme. A.M. was supported by a fellowship of the EMBL International Postdoc programme (EI3POD) under Marie Skłodowska action found [664726]. 

*Conflict of Interest*: none declared. 

## Supplementary Material

btaa682_Supplementary_Data

## References

[btaa682-B1] Becher I. et al (2018) Pervasive protein thermal stability variation during the cell cycle. Cell, 173, 1495–1507.e18. 29706546 10.1016/j.cell.2018.03.053 PMC5998384

[btaa682-B2] Ewing R.M. et al (2007) Large-scale mapping of human protein–protein interactions by mass spectrometry. Mol. Syst. Biol., 3, 89. 17353931 10.1038/msb4100134 PMC1847948

[btaa682-B3] Franken H. et al (2015) Thermal proteome profiling for unbiased identification of direct and indirect drug targets using multiplexed quantitative mass spectrometry. Nat. Protoc., 10, 1567–1593. 26379230 10.1038/nprot.2015.101

[btaa682-B4] Gatto L. et al (2014) A foundation for reliable spatial proteomics data analysis. Mol. Cell. Proteom., 13, 1937–1952. 10.1074/mcp.M113.036350 PMC4125728 24846987

[btaa682-B5] Heusel M. et al (2019) Complex-centric proteome profiling by SEC-SWATH-MS. Mol. Syst. Biol., 15, e8438. 30642884 10.15252/msb.20188438 PMC6346213

[btaa682-B6] Mateus A. et al (2020) Thermal proteome profiling for interrogating protein interactions. Mol. Syst. Biol., 16, e9232. 32133759 10.15252/msb.20199232 PMC7057112

[btaa682-B7] Ori A. et al (2016) Spatiotemporal variation of mammalian protein complex stoichiometries. Genome Biol., 17, 47. 26975353 10.1186/s13059-016-0912-5 PMC4791834

[btaa682-B8] Perrin J. et al (2020) Identifying drug targets in tissues and whole blood with thermal-shift profiling. Nat. Biotechnol., 38, 303–308. 31959954 10.1038/s41587-019-0388-4

[btaa682-B9] Prunuske A.J. et al (2006) Nuclear envelope breakdown is coordinated by both nup358/RanBP2 and nup153, two nucleoporins with zinc finger modules. Mol. Biol. Cell, 17, 760–769. 16314393 10.1091/mbc.E05-06-0485 PMC1356586

[btaa682-B10] Roux K.J. et al (2012) A promiscuous biotin ligase fusion protein identifies proximal and interacting proteins in mammalian cells. J. Cell Biol., 196, 801–810. 22412018 10.1083/jcb.201112098 PMC3308701

[btaa682-B11] Savitski M.M. et al (2014) Tracking cancer drugs in living cells by thermal profiling of the proteome. Science, 346, 1255784. 25278616 10.1126/science.1255784

[btaa682-B12] Tan C.S.H. et al (2018) Thermal proximity coaggregation for systemwide profiling of protein complex dynamics in cells. Science, 359, 1170–1177. 29439025 10.1126/science.aan0346

