# Supplementary material for: Rtpca: an R package for differential thermal proximity coaggregation analysis
Source: Bioinformatics. 2020 Jul 27;37(3):431–3. doi: 10.1093/bioinformatics/btaa682 (PMC8058776; doi:10.1093/bioinformatics/btaa682)
Supplement: btaa682_Supplementary_Data [file btaa682_supplementary_data.pdf]

Supplementary Information: *Rtpca: an R package for differential thermal coaggregation analysis*

Nils Kurzawa[1], **André Mateus**[1], **and Mikhail M. Savitski**[1]

[1]European Molecular Biology Laboratory (EMBL), Genome Biology Unit

18 July, 2020

Package

Rtpca 0.99.8

Contents

1 Introduction 2

2 Step-by-step walk through the data analysis 2

2.1 Getting TPP data into a valid import format 2

2.2 Multiple testing burden in testing for differential PPIs 4

2.3 Complex-centric analysis 5

2.4 PPI-centric analysis 10

3 Comparison of the results obtained by both strategies. 16

4 Conclusion 17

References 18

1 Introduction

Thermal proteome profiling (TPP) (Mateus et al., 2020; Savitski et al., 2014) is a mass spectrometry-based, proteome-wide implemention of the cellular thermal shift assay (Molina et al., 2013). It was originally developed to study drug-(off-)target engagement. However, it was realized that profiles of interacting protein pairs appeared more similar than by chance (Tan et al., 2018) which was coined as 'thermal proximity co-aggregation' (TPCA) (Tan et al., 2018). The R package `Rtpca` enables analysis of TPP datasets using the TPCA concept for studying protein-protein interactions and protein complexes and also allows to test for differential protein-protein interactions (PPIs) across different conditions.
Here, we exemplify the analysis based on a dataset by Becher et al. (2018) which provides temperature range TPP (TPP-TR) experiments for synchronized HeLa cells in G1/S cell cycle stage versus M phase.
Note: The paper by Becher et al. (2018) also includes 2D-TPP (Becher et al., 2016) data which is in general more sensitive to changes in protein abundance or stability. This data can also be informative on dynamics of protein-protein interactions based on correlations analysis of 2D-TPP profiles of annotated interactors. However, the advantage of TPP-TR data is that one can test for coaggregation which, if significant, is directly indicative of protein-protein interaction or complex assembly.

2 Step-by-step walk through the data analysis

First, we need to load the required libraries (these need to be installed as specified in the comments):

```
library(dplyr) # install.packages("dplyr")
library(readxl) # install.packages("readxl")
library(Rtpca) # BiocManager::install("nkurzaw/Rtpca")
library(ggplot2) # install.packages("ggplot2")
library(eulerr) # install.packages("eulerr")
```

Then, we download the supplementary data from Becher et al. (2018) which contains the TPP data which we'll be using:

```
if(!file.exists("1-s2.0-S0092867418303854-mmc4.xlsx")){
    download.file(
        url = "https://ars.els-cdn.com/content/image/1-s2.0-S0092867418303854-mmc4.xlsx",
        destfile = "1-s2.0-S0092867418303854-mmc4.xlsx",
        mode = "wb")
}
```

2.1 Getting TPP data into a valid import format

The ideal input format for using the `Rtpca` package is a list of `ExpressionSets` as obtained by importing the data with the `TPP` package (Franken et al., 2015). We showcase how this can be done in the `Rtpca` package vignette.
However, in order to facilitate usage of the `Rtpca` functions if the raw data of a TPP experiment are not available, such as for the case we are exemplifying here, another supported input

format of the data is a simple list of matrices. In the case replicates are available these can be simply incorporated as different list elements. Here we work with the median fold changes for each condition, thus our list objects will only contain one element each. The rows of the matrices should contain the different measured proteins and the columns the relative soluble fraction at different temperatures measured by TMT reporter ions. Since the column names will often represent the measured TMT channels, the `Rtpca` package additionally expects an `attribute` named `temperature` (shown below how to define, not needed if the input is an object imported with the `TPP` package).

We show here, how such a format can be created based on the example using the supplementary table supplied by Becher et al. (2018):

```
supp_tab_becher_s4 <- read_xlsx("1-s2.0-S0092867418303854-mmc4.xlsx",
                                sheet = "TableS4_TPP-TR")

temperature_anno <-
    as.numeric(
        gsub("T", "", gsub("_.+", "", colnames(
            supp_tab_becher_s4 %>%
                dplyr::select(matches("mean\\.fc"))))))
temperature_anno
##  [1] 37.0 40.4 44.0 46.9 49.8 52.9 55.5 58.6 62.0 66.3
```

We then extract the data for G1/S:

```
g1s_df <- supp_tab_becher_s4 %>%
    filter(cell.cycle == "G1_S") %>%
    dplyr::select(
        gene_name,
        replicates = found.in.reps,
        max_qupm = max.qupm,
        min_qupm = min.qupm,
        matches("mean\\.fc")) %>%
    filter(min_qupm > 3,
           replicates == 3)
```

And we create as matrix, define its row names and supply and attribute vector specifying the temperatures represented by the various TMT channels:

```
g1s_mat <- as.matrix(
    g1s_df %>% dplyr::select(dplyr::matches("mean\\.fc"))
    )
rownames(g1s_mat) <- g1s_df$gene_name
attributes(g1s_mat)$temperature <- temperature_anno
```

This is what the matrix looks like now:

```
head(g1s_mat)
##            T37_mean.fc T40.4_mean.fc T44_mean.fc T46.9_mean.fc T49.8_mean.fc
## A0A0C4DFX4   0.9993307     0.9983367   0.9357640     0.6997003     0.3706639
## AAAS         0.9993307     1.0226134   1.0041548     0.9816410     0.8452729
## AACS         0.9993307     0.9719008   0.7917094     0.5805516     0.4240778
## AAK1         0.9993307     0.9871302   0.9282311     0.8574222     0.6130328
```

```
## AAR2         0.9993307     1.0157685   0.9433393     0.7480540     0.5182360
## AARS         0.9993307     0.9783493   0.9557112     0.9231163     0.7440536
##            T52.9_mean.fc T55.5_mean.fc T58.6_mean.fc T62_mean.fc T66.3_mean.fc
## A0A0C4DFX4     0.2201420     0.1359158    0.09358177  0.06957155    0.05043689
## AAAS           0.6147366     0.2936267    0.12660082  0.07373691    0.05785474
## AACS           0.2602694     0.1729255    0.10649716  0.06847030    0.04615535
## AAK1           0.3170842     0.1465551    0.09536986  0.06222029    0.04243022
## AAR2           0.2603946     0.1264987    0.08088046  0.05285895    0.03565177
## AARS           0.3476423     0.1190308    0.07134315  0.04910556    0.03428983
```

and it's attirbutes

```
summary(attributes(g1s_mat))
##             Length Class  Mode
## dim          2      -none- numeric
## dimnames     2      -none- list
## temperature 10      -none- numeric
```

Now, we do the same for the M phase dataset:

```
m_df <- supp_tab_becher_s4 %>%
    filter(cell.cycle == "M") %>%
    dplyr::select(
        gene_name,
        replicates = found.in.reps,
        max_qupm = max.qupm,
        min_qupm = min.qupm,
        matches("mean\\.fc")) %>%
    filter(min_qupm > 3,
           replicates == 3)
```

```
m_mat <- as.matrix(
    m_df %>% dplyr::select(dplyr::matches("mean\\.fc"))
    )
rownames(m_mat) <- m_df$gene_name
attributes(m_mat)$temperature <- temperature_anno
```

2.2 Multiple testing burden in testing for differential PPIs

In principle, we could now go ahead and test all possible PPIs for differential coaggregation in the datasets acquired in the two different cell cycle phases, however in practise this is not feasible. The reason behind this is that the larger the annotation of PPIs is, the higher will be our multiple testing burden and the less likely we are to identify true positive PPI changes.
Thus, below we suggest two possible strategies that lead to a significant reduction in tests in comparison to e.g. testing all StringDb (Szklarczyk et al., 2019) annotated PPIs above a certain threshold (even though using a very high threshold (990 or even higher) might also be a viable strategy).
The first approach ('complex-centric approach') first tests for coaggregation of protein complexes separately in the different conditions. All PPIs in significantly coaggregating complexes in any of the conditions are then used in a secound step to test for differential coaggregation

across the conditions.
The secound approach ('PPI-centric approach') uses a similar strategy, but tests for significant PPI coaggregation separately in the different conditions and then chooses significant interactions across both conditions for further testing for differential behavior.

2.3 Complex-centric analysis

We start by loading an annotation of mammalian complexes by Ori et al. (2016), which comes with the `Rtpca` package:

```
data("ori_et_al_complexes_df")
ori_et_al_complexes_df
## # A tibble: 2,597 x 3
##    ensembl_id      protein id
##    <chr>           <chr>   <chr>
##  1 ENSG00000222028 PSMB11  26S Proteasome
##  2 ENSG00000108671 PSMD11  26S Proteasome
##  3 ENSG00000197170 PSMD12  26S Proteasome
##  4 ENSG00000110801 PSMD9   26S Proteasome
##  5 ENSG00000115233 PSMD14  26S Proteasome
##  6 ENSG00000101182 PSMA7   26S Proteasome
##  7 ENSG00000108344 PSMD3   26S Proteasome
##  8 ENSG00000101843 PSMD10  26S Proteasome
##  9 ENSG00000165916 PSMC3   26S Proteasome
## 10 ENSG00000008018 PSMB1   26S Proteasome
## # ... with 2,587 more rows
```

The crucial columns it contains are the following `protein`: a column using the same identifiers (Gene names (in this case), Uniprot ids or Ensembl protein ids) as the row names of the supplied input matrix or ExpressionSet object and `id`: unique protein complex ids.

Then, we perform a TPCA analysis based on complexes only in the G1/S condition:

```
G1S_TPCA <- runTPCA(
    objList = list(g1s_mat),
    complexAnno = ori_et_al_complexes_df)
## Checking input arguments.
##
## Creating distance matrices.
##
## Testing for complex co-aggregation.
##
## Performning Complex ROC analysis.
```

We can plot a ROC curve to see how predictive our data is for recovering annotated protein complexes by evoking:

```
plotComplexRoc(G1S_TPCA, computeAUC = TRUE)
```

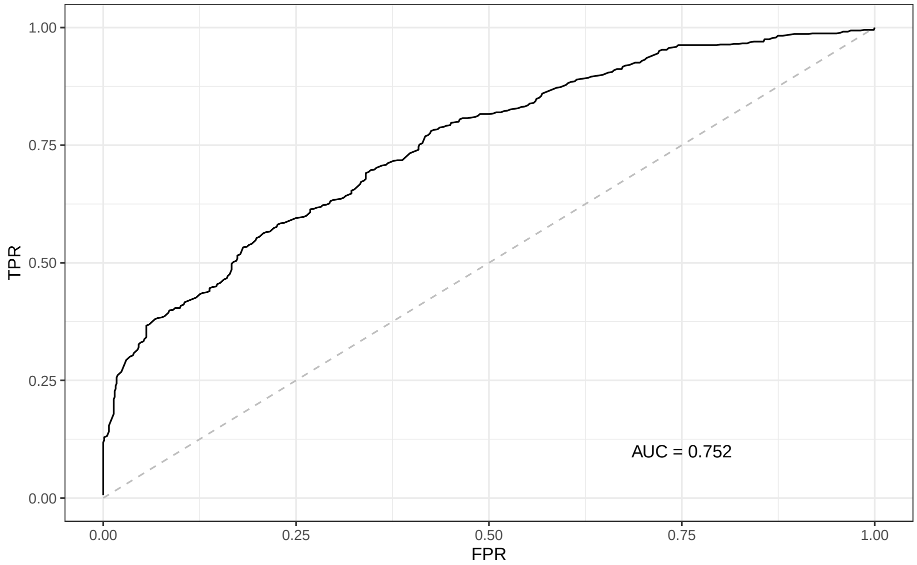

Since it would not be feasible to compare all annotated protein complexes (true positives) with all non-complex annotated groups of proteins (false positives), the function uses several random permutations of the input complex annotation table as a proxy of false positives found by ranking groups of proteins by low average Euclidean melting curve distances. **Note:** This procedure will be slightly different each time it is run, since the permutations of the complex annotation table will be different each time. Thus, it is recommended to set a random number generator seed to get reproducible results.

We can now inspect significantly co-melting protein complexes, like this:

```
tpcaResultTable(G1S_TPCA) %>% filter(p_adj < 0.1)
## # A tibble: 46 x 5
##    complex_name                        count mean_dist  p_value    p_adj
##    <chr>                               <int>     <dbl>    <dbl>    <dbl>
##  1 26S Proteasome                         33    0.441  1.60e- 2 6.78e- 2
##  2 Nuclear pore complex (NPC)             24    0.341  2.22e-16 2.98e-15
##  3 BAF complex                             7    0.214  7.00e- 4 7.51e- 3
##  4 Spliceosome-U2                          9    0.333  1.42e- 2 6.62e- 2
##  5 Anaphase promoting complex (APC)        4    0.0948 2.22e-16 2.98e-15
##  6 multi-tRNAsynthase complex             10    0.123  2.22e-16 2.98e-15
##  7 RNA polymerase III core complex         3    0.189  2.75e- 2 9.69e- 2
##  8 RNA polymerase II core complex          4    0.190  6.30e- 3 3.82e- 2
##  9 COP9 signalosome                        8    0.236  9.00e- 4 8.52e- 3
## 10 MCM complex                             7    0.190  1.00e- 4 1.24e- 3
## # ... with 36 more rows
```

```
g1s_significant_complex_comelting <-
    filter(tpcaResultTable(G1S_TPCA), p_adj < 0.1)$complex_name
```

Next, we perform the same analysis for only the M phase condition:

```
M_TPCA <- runTPCA(
    objList = list(m_mat),
    complexAnno = ori_et_al_complexes_df)
```

```
## Checking input arguments.
##
## Creating distance matrices.
##
## Testing for complex co-aggregation.
##
## Performning Complex ROC analysis.
```

We can see that the predictive performance of this dataset for protein complexes is not quite as good as for the G1/S one, but still pretty decent:

```
plotComplexRoc(M_TPCA, computeAUC = TRUE)
```

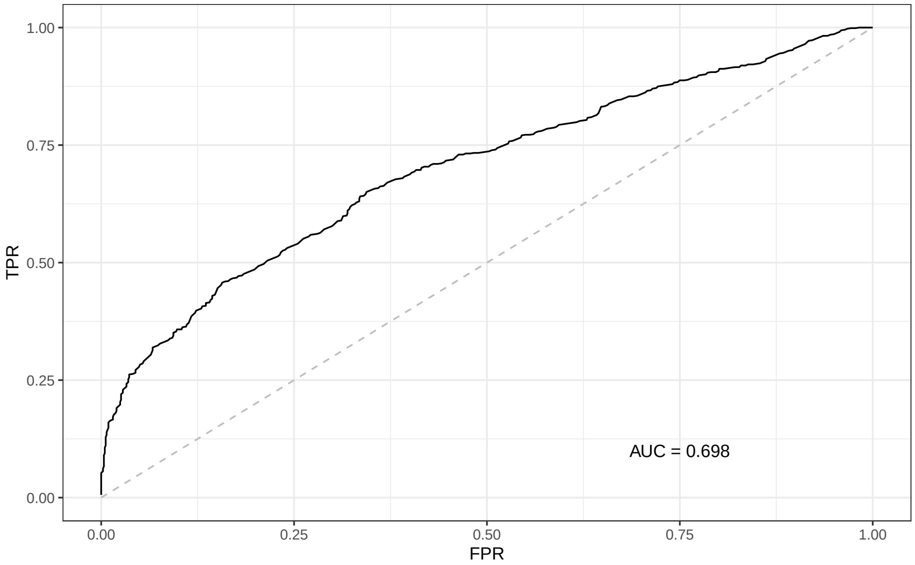


```
tpcaResultTable(M_TPCA) %>% filter(p_adj < 0.1)
## # A tibble: 56 x 5
##    complex_name                          count mean_dist  p_value    p_adj
##    <chr>                                 <int>     <dbl>    <dbl>    <dbl>
##  1 Nuclear pore complex (NPC)               25    0.332  8.00e- 4 9.77e- 3
##  2 BAF complex                               9    0.140  2.22e-16 4.75e-15
##  3 Integrator                                7    0.254  9.70e- 3 4.74e- 2
##  4 NuRD complex                              9    0.254  4.30e- 3 2.54e- 2
##  5 Anaphase promoting complex (APC)          4    0.166  1.12e- 2 5.18e- 2
##  6 Cohesin complex                           7    0.245  8.50e- 3 4.38e- 2
##  7 Transcription-export (TREX) complex       8    0.312  2.79e- 2 8.52e- 2
##  8 multi-tRNAsynthase complex               10    0.0811 2.22e-16 4.75e-15
##  9 RANBP9-containing complex                 3    0.0898 3.00e- 3 1.97e- 2
## 10 RNA polymerase II core complex            4    0.174  1.35e- 2 5.37e- 2
## # ... with 46 more rows
```

Based on the protein complexes which we find significantly assembled in either condition, we will select the protein-protein interactions to test for in a differential TPCA:

```
m_significant_complex_comelting <-
    filter(tpcaResultTable(M_TPCA), p_adj < 0.1)$complex_name

all_significant_complex_comelting <-
    unique(c(g1s_significant_complex_comelting,
             m_significant_complex_comelting))
```

We load the annotation of protein-protein interactions within complexes that is composed of PPIs from StringDb (Szklarczyk et al., 2019) and the complex annotation by Ori et al. (2016) and filter it for protein complexes that we have seen to coaggregate in the analysis above.

```
data("ori_et_al_complex_ppis")

filtered_complex_ppis <- ori_et_al_complex_ppis %>%
    filter(complex_name %in% all_significant_complex_comelting)
```

We now run the differential TPCA by evoking:

```
M_vs_G1S_diff_TPCA <- runDiffTPCA(
    objList = list(g1s_mat),
    contrastList = list(m_mat),
    ctrlCondName = "G1/S",
    contrastCondName = "M",
    ppiAnno = filtered_complex_ppis,
    n = 10^6
)
## Checking input arguments.
## Creating distance matrices.
## Comparing annotated protein-pairs across conditions.
## Comparing random protein-pairs across conditions.
## Generating result table.
```

This analysis gives us a `tpcaResult` object:

```
M_vs_G1S_diff_TPCA
## class: tpcaResult
## Slot "ObjList": of class list and length 1
## Slot "ContrastList": of class list and length 1
## Slot "DistMat" with dimension: 2658 2658
## Slot "ContrastDistMat" with dimension: 3086 3086
## Slot "ComplexAnnotation" of class: data.frame with dim: 0 0
## Slot "ComplexBackgroundDistributionList" of class: list with length: 0
## Slot "PPiAnnotation" of class: tbl_df tbl data.frame with dim: 4466 5
## Slot "PPiRocTable" of class: data.frame with dim: 0 0
## Slot "PPiRocTableAnno" of class: data.frame with dim: 0 0
## Slot "ComplexRocTable" of class: data.frame with dim: 0 0
## Slot "summaryMethod": median
## Slot "distMethod": euclidean
## Slot "tpcaResultTable" of class: data.frame with dim: 0 0
## Slot "diffTpcaResultTable" of class: tbl_df tbl data.frame with dim: 1549 10
```

We can now plot the result in form of a volcano plot:

```r
plotDiffTpcaVolcano(M_vs_G1S_diff_TPCA,
                    setXLim = TRUE,
                    xlimit = c(-0.75, 0.75))
```

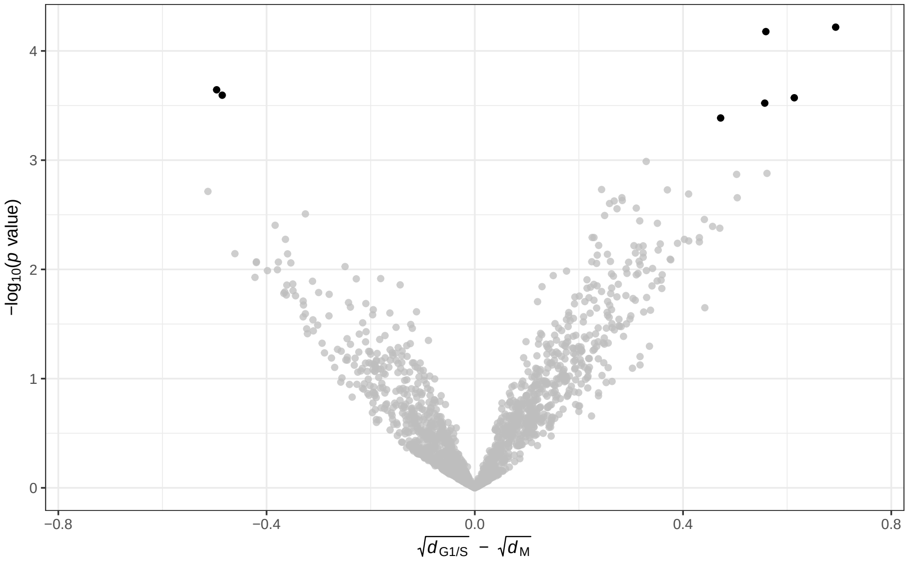

We can now inspect the significant results:

```r
diffTpcaResultTable(M_vs_G1S_diff_TPCA) %>%
    dplyr::select(pair, rssC1_rssC2, p_value, p_adj) %>%
    arrange(p_value)
## # A tibble: 1,549 x 4
##    pair           rssC1_rssC2   p_value  p_adj
##    <chr>                <dbl>     <dbl>  <dbl>
##  1 NUP205:RANBP2       0.834  0.0000606 0.0515
##  2 NUP88:RANBP2        0.357  0.0000665 0.0515
##  3 RPS6:RPSA          -0.279  0.000227  0.0776
##  4 RPS23:RPSA         -0.263  0.000254  0.0776
##  5 NUP93:RANBP2        0.679  0.000268  0.0776
##  6 NUP188:RANBP2       0.470  0.000300  0.0776
##  7 AAAS:TPR            0.260  0.000411  0.0909
##  8 PSMB1:PSMB4         0.0786 0.00103   0.199
##  9 NUP54:RANBP2        0.710  0.00132   0.203
## 10 NUP107:RANBP2       0.461  0.00135   0.203
## # ... with 1,539 more rows
```

To validate significant PPIs we can inspect their melting curves:

```r
plotPPiProfiles(M_vs_G1S_diff_TPCA, pair = c("NUP205", "RANBP2"))
```

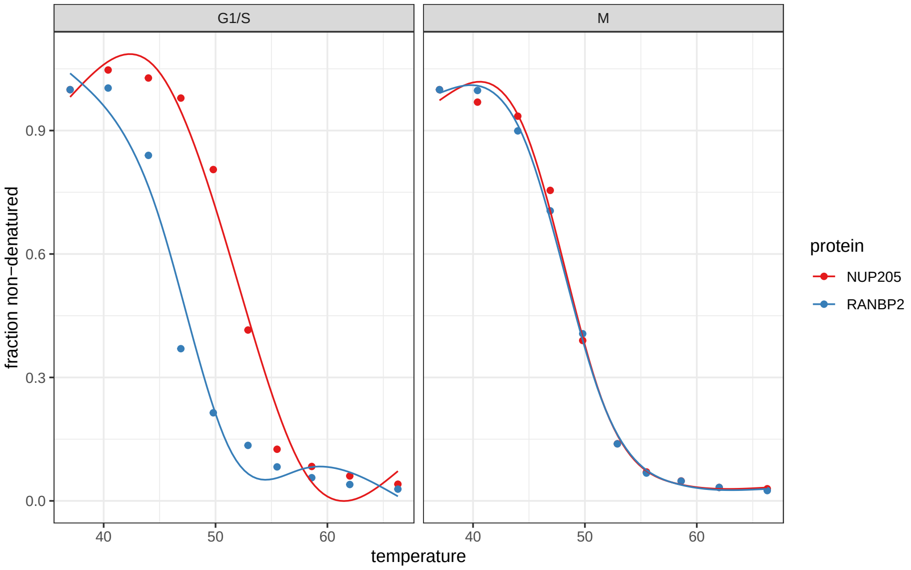


```r
plotPPiProfiles(M_vs_G1S_diff_TPCA, pair = c("RPS6", "RPSA"))
```

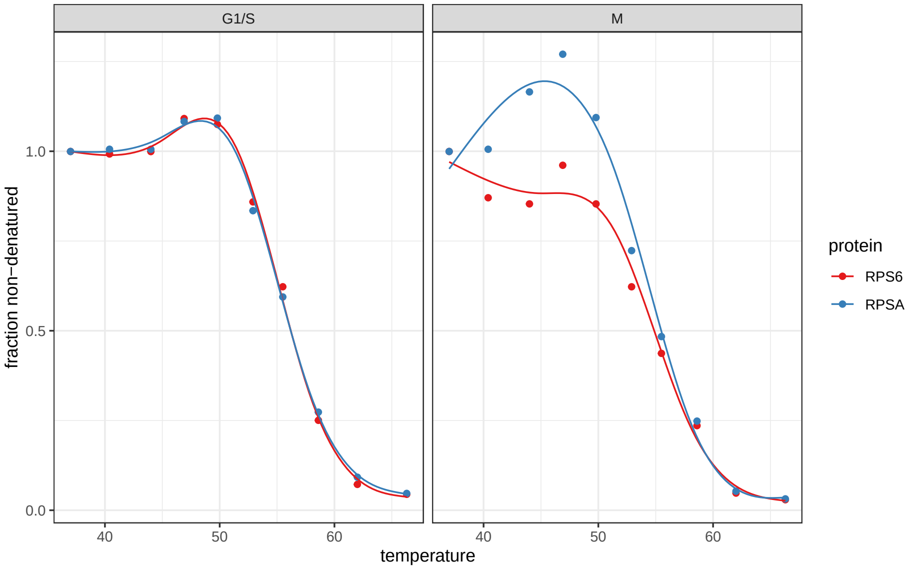

2.4 PPI-centric analysis

For the PPI-centric analysis, we first load PPIs annotated by StringDb (Szklarczyk et al., 2019):

```r
data("string_ppi_df")
string_ppi_df
## # A tibble: 252,558 x 4
##    x     y       combined_score pair
##    <chr> <chr>            <dbl> <chr>
##  1 ARF5  SPTBN2             909 ARF5:SPTBN2
```

```
##   2 ARF5  KIF13B              910 ARF5:KIF13B
##   3 ARF5  KIF21A              910 ARF5:KIF21A
##   4 ARF5  TMED7               906 ARF5:TMED7
##   5 ARF5  ARFGAP1             971 ARF5:ARFGAP1
##   6 ARF5  ANK2                915 ANK2:ARF5
##   7 ARF5  KLC1                905 ARF5:KLC1
##   8 ARF5  COPZ2               927 ARF5:COPZ2
##   9 ARF5  KIF15               914 ARF5:KIF15
## 10 ARF5  DCTN5               902 ARF5:DCTN5
## # ... with 252,548 more rows
```

This table has been created from the human *protein.links* table downloaded from the StringDb website. It can serve as a template for users to create equivalent tables for other organisms. Its essential columns are `x`: 1st interactor, `y`: 2nd interactor and `pair`: unique id of both interactors in alphabetical order.

And we filter this table to only contain high confidence PPIs

```
string_ppi_975_df <- string_ppi_df %>%
    filter(combined_score >= 975)
```

Then we start our analysis based on PPIs:

```
G1S_PPI_TPCA <- runTPCA(
    objList = list(g1s_mat),
    ppiAnno = string_ppi_975_df,
    nSamp = 10^6)
## Checking input arguments.
##
## Creating distance matrices.
##
## Testing for complex co-aggregation.
##
## Performing PPi ROC analysis.
```

As for the complex-centric analysis we get back a `tpcaResult` object:

```
G1S_PPI_TPCA
## class: tpcaResult
## Slot "ObjList": of class list and length 1
## Slot "ContrastList": of class list and length 0
## Slot "DistMat" with dimension: 2658 2658
## Slot "ContrastDistMat" with dimension: 0 0
## Slot "ComplexAnnotation" of class: tbl_df tbl data.frame with dim: 16460 3
## Slot "ComplexBackgroundDistributionList" of class: list with length: 1
## Slot "PPiAnnotation" of class: tbl_df tbl data.frame with dim: 39176 4
## Slot "PPiRocTable" of class: tbl_df tbl data.frame with dim: 3531153 3
## Slot "PPiRocTableAnno" of class: tbl_df tbl data.frame with dim: 3531153 2
## Slot "ComplexRocTable" of class: data.frame with dim: 0 0
## Slot "summaryMethod": median
## Slot "distMethod": euclidean
## Slot "tpcaResultTable" of class: tbl_df tbl data.frame with dim: 8230 5
```

```
## Slot "diffTpcaResultTable" of class: data.frame with dim: 0 0
```

And we can also inspect a ROC curve for this analysis:

```
plotPPiRoc(G1S_PPI_TPCA, computeAUC = TRUE)
```

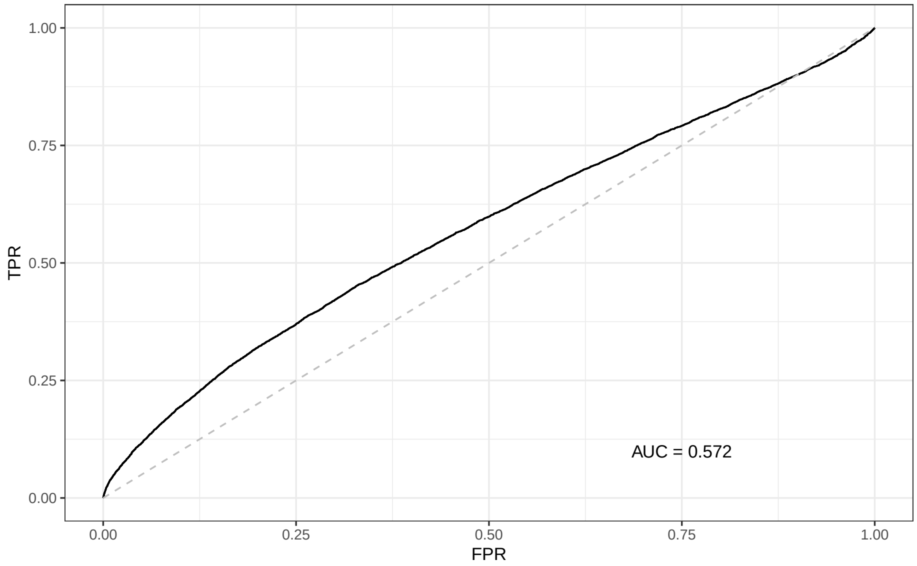

To inspect which PPIs coaggregated significantly, we can evoke:

```
tpcaResultTable(G1S_PPI_TPCA) %>% filter(p_adj < 0.1) %>% arrange(p_value)
## # A tibble: 77 x 5
##    complex_name  count mean_dist  p_value    p_adj
##    <chr>         <int>     <dbl>    <dbl>    <dbl>
##  1 CANX:GANAB        2    0.0298 2.22e-16 1.83e-13
##  2 EIF3D:EIF3E       2    0.0165 2.22e-16 1.83e-13
##  3 ERLIN1:ERLIN2     2    0.0301 2.22e-16 1.83e-13
##  4 HADHA:HADHB       2    0.0300 2.22e-16 1.83e-13
##  5 HSPA1A:HSPA1B     2    0      2.22e-16 1.83e-13
##  6 IARS:LARS         2    0.0266 2.22e-16 1.83e-13
##  7 IARS:RARS         2    0.0227 2.22e-16 1.83e-13
##  8 LAMA5:LAMC1       2    0.0262 2.22e-16 1.83e-13
##  9 MCM2:MCM4         2    0.0220 2.22e-16 1.83e-13
## 10 SAE1:UBA2         2    0.0312 2.22e-16 1.83e-13
## # ... with 67 more rows
```

And we can run the same analysis for the M-phase dataset:

```
M_PPI_TPCA <- runTPCA(
    objList = list(m_mat),
    ppiAnno = string_ppi_975_df,
    nSamp = 10^6)
## Checking input arguments.
##
## Creating distance matrices.
##
```

```
## Testing for complex co-aggregation.
##
## Performing PPi ROC analysis.
```

```
plotPPiRoc(M_PPI_TPCA, computeAUC = TRUE)
```

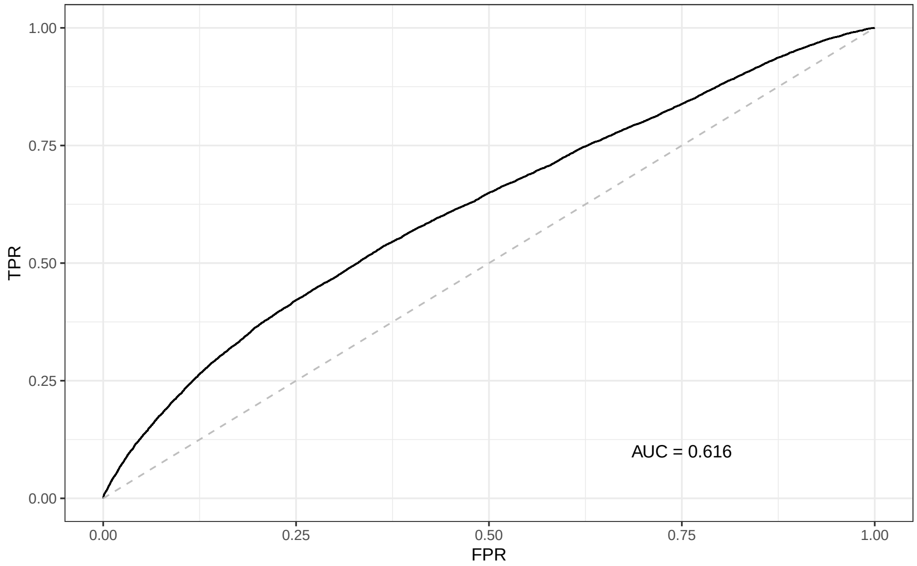

Note: The AUC for recovering PPIs using the TPCA approach is usually lower than for recovering protein complexes. This is due to the fact that it is less likely to find three or more proteins showing similar melting curves by chance (protein complex analysis), than it is for two proteins (PPI-based analysis).

```
tpcaResultTable(M_PPI_TPCA) %>% filter(p_adj < 0.1) %>% arrange(p_value)
## # A tibble: 18 x 5
##    complex_name      count mean_dist  p_value    p_adj
##    <chr>             <int>     <dbl>    <dbl>    <dbl>
##  1 AP2A1:AP2M1           2    0.0198 2.22e-16 3.23e-13
##  2 CYFIP1:NCKAP1         2    0.0188 2.22e-16 3.23e-13
##  3 HADHA:HADHB           2    0.0198 2.22e-16 3.23e-13
##  4 HSP90AA1:HSP90AB1     2    0.0230 2.22e-16 3.23e-13
##  5 HSPA1A:HSPA1B         2    0      2.22e-16 3.23e-13
##  6 NAE1:UBA3             2    0.0127 2.22e-16 3.23e-13
##  7 NCAPD2:SMC4           2    0.0218 2.22e-16 3.23e-13
##  8 AIMP1:RARS            2    0.0283 1.00e- 4 5.65e- 2
##  9 CDK1:NCAPG            2    0.0275 1.00e- 4 5.65e- 2
## 10 EPRS:LARS             2    0.0260 1.00e- 4 5.65e- 2
## 11 GTF3C1:GTF3C3         2    0.0281 1.00e- 4 5.65e- 2
## 12 IARS:QARS             2    0.0268 1.00e- 4 5.65e- 2
## 13 MRPL16:MRPL37         2    0.0276 1.00e- 4 5.65e- 2
## 14 NDC80:NUF2            2    0.0250 1.00e- 4 5.65e- 2
## 15 QARS:YARS             2    0.0280 1.00e- 4 5.65e- 2
## 16 RBM17:U2SURP          2    0.0285 1.00e- 4 5.65e- 2
## 17 SMC2:SMC4             2    0.0274 1.00e- 4 5.65e- 2
## 18 SPTAN1:SPTBN1         2    0.0269 1.00e- 4 5.65e- 2
```

By now combining the significantly found coaggregating PPIs (we are a bit less stringent on the adjusted p-value filter here to not reduce the space of possible differential PPIs too strongly), we can define a list of PPIs which we can use to test for differential PPIs across the two cell cycle phases:

```
ppis_to_test_diff <- unique(
    c(filter(G1S_PPI_TPCA@tpcaResultTable, p_adj < 0.2)$complex_name,
      filter(M_PPI_TPCA@tpcaResultTable, p_adj < 0.2)$complex_name)
)

filtered_string_ppis <- string_ppi_975_df %>%
    filter(pair %in% ppis_to_test_diff)
```

Based on these PPIs we can now again run a differential TPCA:

```
M_vs_G1S_PPI_diff_TPCA <- runDiffTPCA(
    objList = list(g1s_mat),
    contrastList = list(m_mat),
    ctrlCondName = "G1/S",
    contrastCondName = "M",
    ppiAnno = filtered_string_ppis,
    n = 10^6
)
## Checking input arguments.
## Creating distance matrices.
## Comparing annotated protein-pairs across conditions.
## Comparing random protein-pairs across conditions.
## Generating result table.
```

```
M_vs_G1S_PPI_diff_TPCA <- readRDS("prerun/M_vs_G1S_PPI_diff_TPCA.rds")
```

```
plotDiffTpcaVolcano(M_vs_G1S_PPI_diff_TPCA,
                    setXLim = TRUE,
                    xlimit = c(-0.75, 0.75))
```

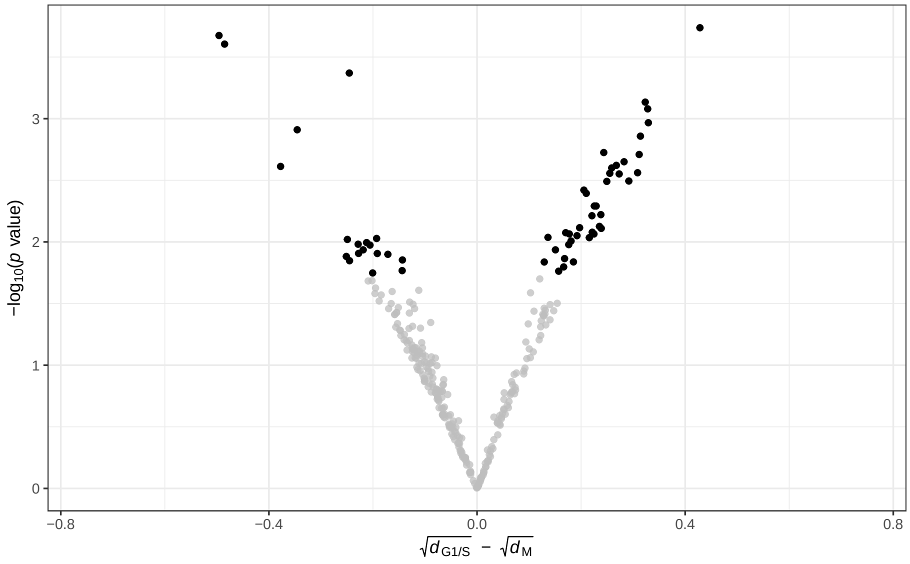

Again, we can now inspect the significant results:

```
diffTpcaResultTable(M_vs_G1S_PPI_diff_TPCA) %>%
    dplyr::select(pair, rssC1_rssC2, p_value, p_adj) %>%
    arrange(p_value)
## # A tibble: 304 x 4
##    pair         rssC1_rssC2  p_value  p_adj
##    <chr>              <dbl>    <dbl>  <dbl>
##  1 CDC5L:EXOC7       0.152  0.000183 0.0252
##  2 RPS6:RPSA        -0.279  0.000211 0.0252
##  3 RPS23:RPSA       -0.263  0.000248 0.0252
##  4 EIF3D:EIF3E      -0.0192 0.000426 0.0324
##  5 RPL10A:RPS16      0.0666 0.000734 0.0422
##  6 CDC73:RNF20       0.0727 0.000833 0.0422
##  7 PSMB1:PSMB4       0.0786 0.00108  0.0468
##  8 PPID:PTGES3      -0.0991 0.00123  0.0468
##  9 CTR9:SUPT6H       0.0699 0.00139  0.0468
## 10 AIMP1:RARS        0.0279 0.00188  0.0474
## # ... with 294 more rows
```

And again we plot some of the significantly differentially coaggregating protein pairs:

```
plotPPiProfiles(M_vs_G1S_PPI_diff_TPCA, c("CDC5L", "EXOC7"))
```

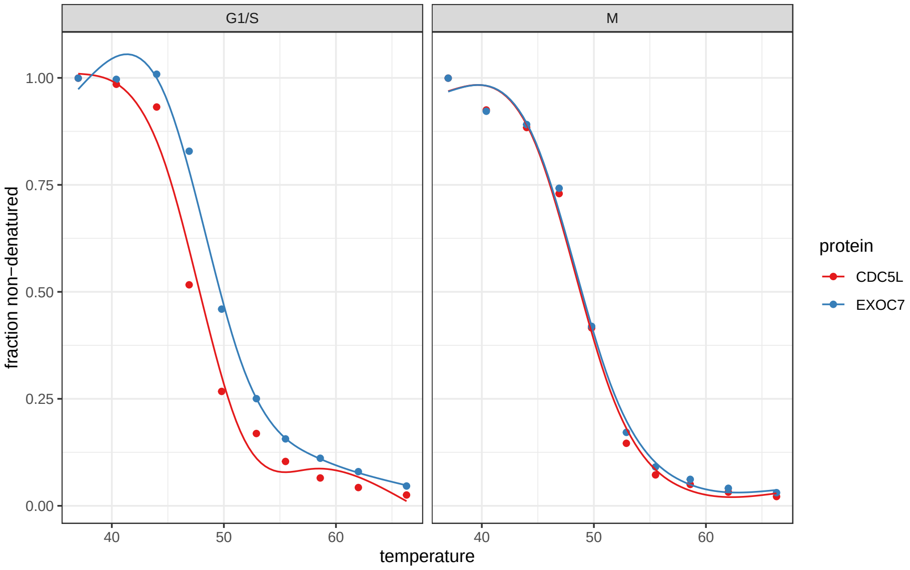


```
plotPPiProfiles(M_vs_G1S_PPI_diff_TPCA, c("EIF3D", "EIF3E"))
```

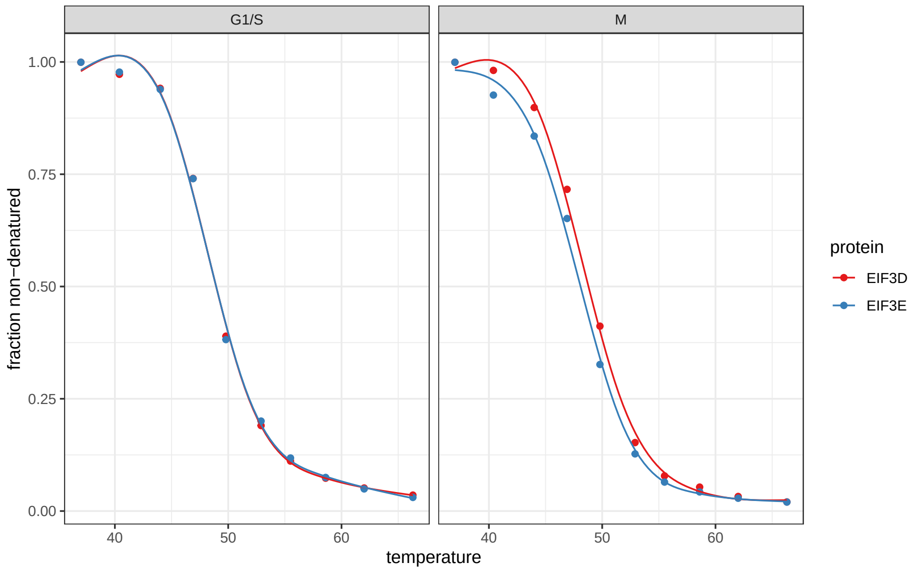


```
plotPPiProfiles(M_vs_G1S_PPI_diff_TPCA, c("CCNT1", "CDK9"))
```

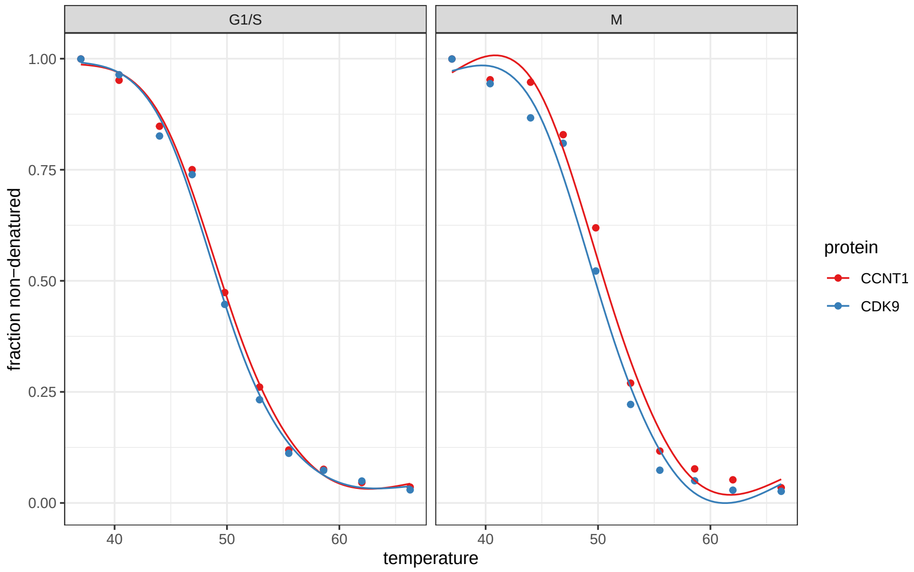

3 Comparison of the results obtained by both strategies

In order to asses how many differential PPIs can be recovered with either of the approaches, we plot a Venn diagram below:

```
complex_centric_hits <- diffTpcaResultTable(M_vs_G1S_diff_TPCA) %>%
    dplyr::select(pair, rssC1_rssC2, p_value, p_adj) %>%
    arrange(p_value) %>%
    filter(p_adj < 0.1)
```

```
ppi_centric_hits <- diffTpcaResultTable(M_vs_G1S_PPI_diff_TPCA) %>%
    dplyr::select(pair, rssC1_rssC2, p_value, p_adj) %>%
    arrange(p_value) %>%
    filter(p_adj < 0.1)

all_hits <- union(complex_centric_hits$pair,
                  ppi_centric_hits$pair)

venn_df <- data.frame(
  complex_centric = all_hits %in% complex_centric_hits$pair,
  ppi_centric = all_hits %in% ppi_centric_hits$pair
)

plot(venn(venn_df))
```

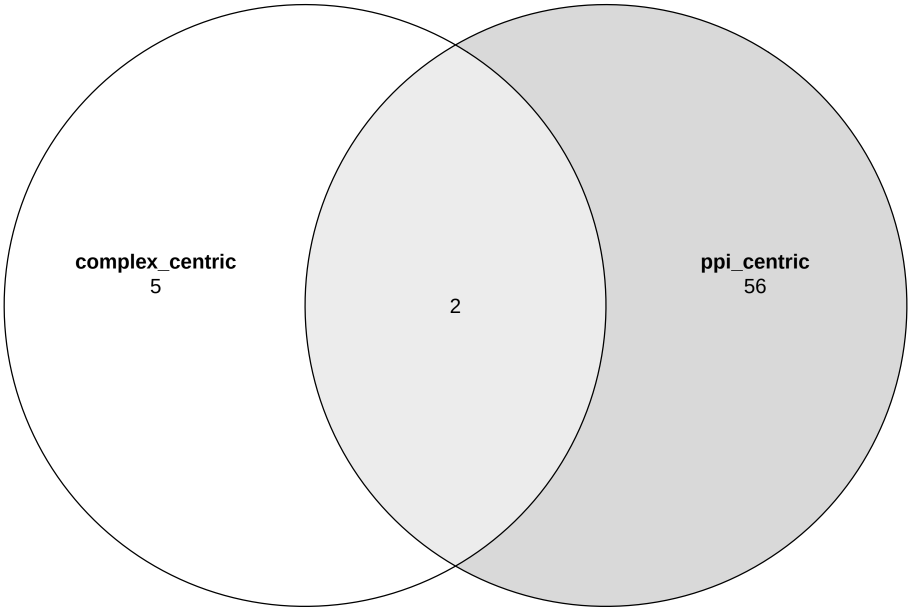

It appears that both approaches pick up a set of distinct differential PPIs. The PPI-centric approach appears to recover more significant PPI changes, however the complex-centric one reveals more intra-complex centered interactions changes.

4 Conclusion

`Rtpca` offers user-friendly exploration of TPP datasets for PPIs and allows to assess significantly changing PPIs across different conditions. We exemplify here, how this can be done using the TPP dataset of different phases of the human cell cycle (Becher et al., 2018) from which we recover several differentially coaggregating protein pairs which are known to change during these phases.
A challenge in the analysis remains the high numbers of hypothesis tests that have to be performed which require multiple testing adjustment and are limited in sensitivity. In the

future, methods such as *independent hypothesis weighting* (`IHW`) (Ignatiadis et al., 2016), exploiting covariates such as number of unique peptides used for quantification, could be used to circumvent this problem and further improve sensitivity of the approach.

```
sessionInfo()
## R version 4.0.0 Patched (2020-05-04 r78358)
## Platform: x86_64-apple-darwin17.0 (64-bit)
## Running under: macOS Mojave 10.14.6
##
## Matrix products: default
## BLAS:   /Library/Frameworks/R.framework/Versions/4.0/Resources/lib/libRblas.dylib
## LAPACK: /Library/Frameworks/R.framework/Versions/4.0/Resources/lib/libRlapack.dylib
##
## locale:
## [1] en_US.UTF-8/en_US.UTF-8/en_US.UTF-8/C/en_US.UTF-8/en_US.UTF-8
##
## attached base packages:
## [1] stats     graphics  grDevices utils     datasets  methods   base
##
## other attached packages:
## [1] eulerr_6.1.0     ggplot2_3.3.2    Rtpca_0.99.8     tidyr_1.1.0
## [5] readxl_1.3.1     dplyr_1.0.0      BiocStyle_2.16.0
##
## loaded via a namespace (and not attached):
##  [1] tidyselect_1.1.0    xfun_0.14           purrr_0.3.4
##  [4] splines_4.0.0       lattice_0.20-41     colorspace_1.4-1
##  [7] vctrs_0.3.0         generics_0.0.2      htmltools_0.4.0
## [10] yaml_2.2.1          mgcv_1.8-31         utf8_1.1.4
## [13] rlang_0.4.6         pillar_1.4.4        glue_1.4.1
## [16] withr_2.2.0         BiocGenerics_0.34.0 RColorBrewer_1.1-2
## [19] lifecycle_0.2.0     plyr_1.8.6          stringr_1.4.0
## [22] munsell_0.5.0       gtable_0.3.0        cellranger_1.1.0
## [25] evaluate_0.14       labeling_0.3        Biobase_2.48.0
## [28] knitr_1.28          parallel_4.0.0      fdrtool_1.2.15
## [31] fansi_0.4.1         Rcpp_1.0.4.6        scales_1.1.1
## [34] BiocManager_1.30.10 farver_2.0.3        digest_0.6.25
## [37] stringi_1.4.6       bookdown_0.19       polyclip_1.10-0
## [40] grid_4.0.0          cli_2.0.2           tools_4.0.0
## [43] magrittr_1.5        tibble_3.0.1        crayon_1.3.4
## [46] pkgconfig_2.0.3     ellipsis_0.3.1      Matrix_1.2-18
## [49] polylabelr_0.2.0    pROC_1.16.2         assertthat_0.2.1
## [52] rmarkdown_2.2       R6_2.4.1            nlme_3.1-148
## [55] compiler_4.0.0
```

References

Becher, I., Werner, T., Doce, C., Zaal, E.A., Tögel, I., Khan, C.A., Rueger, A., Muelbaier, M., Salzer, E., Berkers, C.R., et al. (2016). Thermal profiling reveals phenylalanine hydroxylase as an off-target of panobinostat. Nature Chemical Biology *12*, 908–910.

Becher, I., Andrés-Pons, A., Romanov, N., Stein, F., Schramm, M., Baudin, F., Helm, D., Kurzawa, N., Mateus, A., Mackmull, M.-T., et al. (2018). Pervasive Protein Thermal Stability Variation during the Cell Cycle. Cell 1–13.

Franken, H., Mathieson, T., Childs, D., Sweetman, G.M.A., Werner, T., Tögel, I., Doce, C., Gade, S., Bantscheff, M., Drewes, G., et al. (2015). Thermal proteome profiling for unbiased identification of direct and indirect drug targets using multiplexed quantitative mass spectrometry. Nature Protocols *10*, 1567–1593.

Ignatiadis, N., Klaus, B., Zaugg, J.B., and Huber, W. (2016). Data-driven hypothesis weighting increases detection power in genome-scale multiple testing. Nature Methods *13*, 577–580.

Mateus, A., Kurzawa, N., Becher, I., Sridharan, S., Helm, D., Stein, F., Typas, A., and Savitski, M.M. (2020). Thermal proteome profiling for interrogating protein interactions. Molecular Systems Biology 1–11.

Molina, D.M., Jafari, R., Ignatushchenko, M., Seki, T., Larsson, E.A., Dan, C., Sreekumar, L., Cao, Y., and Nordlund, P. (2013). Monitoring Drug Target Engagement in Cells and Tissues Using the Cellular Thermal Shift Assay. Science *341*, 84–88.

Ori, A., Iskar, M., Buczak, K., Kastritis, P., Parca, L., Andrés-pons, A., Singer, S., Bork, P., and Beck, M. (2016). Spatiotemporal variation of mammalian protein complex stoichiometries. Genome Biology 1–15.

Savitski, M.M., Reinhard, F.B.M., Franken, H., Werner, T., Savitski, M.F., Eberhard, D., Martinez Molina, D., Jafari, R., Dovega, R.B., Klaeger, S., et al. (2014). Tracking cancer drugs in living cells by thermal profiling of the proteome. Science *346*, 1255784.

Szklarczyk, D., Gable, A.L., Lyon, D., Junge, A., Wyder, S., Huerta-Cepas, J., Simonovic, M., Doncheva, N.T., Morris, J.H., Bork, P., et al. (2019). STRING v11: Protein-protein association networks with increased coverage, supporting functional discovery in genome-wide experimental datasets. Nucleic Acids Research *47*, D607–D613.

Tan, C.S.H., Go, K.D., Bisteau, X., Dai, L., Yong, C.H., Prabhu, N., Ozturk, M.B., Lim, Y.T., Sreekumar, L., Lengqvist, J., et al. (2018). Thermal proximity coaggregation for system-wide profiling of protein complex dynamics in cells. Science *0346*, 1–12.
